# Sustainable Use of Sewage Sludge as a Casing Material for Button Mushroom (*Agaricus bisporus*) Cultivation: Experimental and Prediction Modeling Studies for Uptake of Metal Elements

**DOI:** 10.3390/jof8020112

**Published:** 2022-01-25

**Authors:** Pankaj Kumar, Vinod Kumar, Bashir Adelodun, Dalibor Bedeković, Ivica Kos, Ivan Širić, Saad A. M. Alamri, Sulaiman A. Alrumman, Ebrahem M. Eid, Sami Abou Fayssal, Madhumita Goala, Ashish Kumar Arya, Archana Bachheti, Kyung Sook Choi, Fidelis Odedishemi Ajibade, Luis F. O. Silva

**Affiliations:** 1 Agro-Ecology and Pollution Research Laboratory, Department of Zoology and Environmental Science, Gurukula Kangri (Deemed to Be University), Haridwar 249404, India; rs.pankajkumar@gkv.ac.in; 2 Department of Agricultural and Biosystems Engineering, University of Ilorin, Ilorin PMB 1515, Nigeria; adelodun.b@unilorin.edu.ng; 3 Department of Agricultural Civil Engineering, Kyungpook National University, Daegu 41566, Korea; ks.choi@knu.ac.kr; 4 Faculty of Agriculture, University of Zagreb, Svetosimunska 25, 10000 Zagreb, Croatia; dbedekovic@agr.hr (D.B.); ikos@agr.hr (I.K.); isiric@agr.hr (I.Š.); 5 Biology Department, College of Science, King Khalid University, Abha 61321, Saudi Arabia; saralomari@kku.edu.sa (S.A.M.A.); salrumman@kku.edu.sa (S.A.A.); ebrahem.eid@sci.kfs.edu.eg (E.M.E.); 6 Botany Department, Faculty of Science, Kafrelsheikh University, Kafr El-Sheikh 33516, Egypt; 7 Department of Agronomy, Faculty of Agronomy, University of Forestry, 10 Kliment Ohridski Blvd, 1797 Sofia, Bulgaria; sami.aboufaycal@st.ul.edu.lb; 8 Department of Plant Production, Faculty of Agriculture, Lebanese University, Beirut 1302, Lebanon; 9 Nehru College, Pailapool, Affiliated Assam University, Silchar 788098, India; madhumitagoalap@gmail.com; 10 Department of Environment Science, Graphic Era (Deemed to be University), Dehradun 248002, India; ashishtyagi.gkv@gmail.com (A.K.A.); bachheti.archana@gmail.com (A.B.); 11 Department of Civil and Environmental Engineering, Federal University of Technology, Akure PMB 704, Nigeria; foajibade@futa.edu.ng; 12 Key Laboratory of Urban Pollutant Conversion, Institute of Urban Environment, Chinese Academy of Sciences, Xiamen 361021, China; 13 Department of Civil and Environmental Engineering, Universidad de la Costa, Calle 58 #55-66, Barranquilla 080002, Colombia; lsilva8@cuc.edu.co

**Keywords:** bioaccumulation, mushroom cultivation, prediction models, regression analysis, waste management

## Abstract

The present study focused on the use of sewage sludge (SS) as a casing material amendment and the potential uptake of metal elements by the cultivated white button (*Agaricus bisporus*: MS-39) mushroom. Laboratory experiments were performed under controlled environmental conditions to grow *A. bisporus* on the composted wheat straw substrate for 50 days. Different treatments (0, 50, 100, 150, and 200 g/kg) of casing material were prepared by mixing garden and dried SS and applied on the mushroom substrate after proper sterilization. The results revealed that SS application was significant (*p* < 0.05) in accelerating mushroom yield with a biological efficiency of 65.02% for the mixing rate of 200 g/kg. Moreover, the maximum bioaccumulation of selected metal elements (Cu, Cr, Cd, Fe, Mn, and Zn) was observed using the same treatment. Additionally, the multiple regression models constructed for the uptake prediction of metal elements showed an acceptable coefficient of determination (*R*^2^ > 0.9900), high model efficiency (ME > 0.98), and low root mean square error (RMSE < 0.410) values, respectively. The findings of this study represent sustainable use of SS for the formulation of mushroom casing material contributing toward synergistic agro-economy generation and waste management.

## 1. Introduction

Over the last decades, the generation of sewage sludge (SS) has increased dramatically in India because of the increasing population and unplanned urbanization. Historically, SS disposal was considered a secondary issue that was facing humanity [1], but it has been causing environmental pollution with dangerous impacts on all forms of life [2,3]. Sewage sludge disposal should be correctly managed especially in countries with high population density [4,5]. In India, numerous sewage wastewater (SW) treatment plants (around 234 facilities) were built along the banks of major rivers [6]. However, it still could not cope with the daily treatment capacity of SW, estimated at 62 thousand of million liters per day in urban areas. Recent reports state that the majority of the SS is improperly disposed of, with only a small volume being recycled in a sustainable manner [7].

The nutritional quality of commercially produced mushrooms is strongly determined by the chemical composition of the growing substrate [8,9,10,11,12,13,14,15] and the type of supplements added to them [12,14]. *Agaricus bisporus*, commonly known as the white button mushroom, is cultivated on a fermented substrate composed of a mixture of lignocellulosic materials, mainly horse and chicken manure [8,16,17]. During the production process of this mushroom, the casing is essential to stimulate fruit body formation. Casing act as an important layer for mushroom as it assists the carpophores in several ways, such as: providing a physical support system, acting as a water reservoir, assisting in nutrient exchange, osmotic pressure regulation, creating an aerated environment for efficient metabolism, stimulating fructification, facilitating gas interchanges, and providing zones for ion exchange [7]. The casing material used for this aim should essentially provide suitable physicochemical and biological properties that stimulate the process of pinhead formation [18,19]. A suitable casing soil is characterized by a water holding capacity above 45%, porosity above 50%, pH around 7.8, and electrical conductivity below 1.6 mhos [20]. Loam soil (surface layer organic soil) is the most widely used casing material on a commercial scale in India [21]. Nevertheless, various agro-industrial residues have been used as alternatives or as additives to loam soil, such as composted vine shoots [22], spent mushroom substrate and coconut fiber pith [23], waste paper [24,25], and many others [26,27,28].

The use of SS in *Agaricus bisporus* production was first reported by Block [29], who recognized this waste as a good source of nitrogen, vitamins, and trace metals for microorganisms involved in the compost, without affecting (when found in low concentrations) the composition of mushroom carpophores in terms of trace elements [30]. Later, Kāposts et al. [31] noted that mushroom composts amended with sludge had a 20–50% richer but admissible metal elements profile. More recently, Yamauchi et al. [32] found that the use of SS in a compost mixture with cow manure increased the yield and free amino acids content of mushrooms. Commonly, SS is known as valuable organic manure rich in recycled nitrogen and phosphorus [33,34] and is characterized by a relatively low C:N ratio, ranging between 6 and 9 [35,36], high bulk density, and low air-filled porosity [37]. However, reports on the use of SS at the level of casing during *A. bisporus* production processes are almost lacking. Similarly, the impact of this waste type on the metal element profile of mushrooms was mostly reported on those collected from the wild. The carpophores had high concentrations of Cd, Pb, and Hg adjacent to landfill sites of SS [38] and normal As, Cu, Se, and Zn contents in forests subjected to sludge application [30].

Therefore, the current study investigates the use of SS in mixtures with loam soil as a casing material for the production of *A. bisporus* and reports its effect on the yield. Furthermore, the potential risk associated with metal element uptake by the harvested mushrooms was investigated using bioaccumulation and multiple linear regression approaches.

## 2. Materials and Methods

### 2.1. Experimental Materials

A commercial strain of the white button (*Agaricus bisporus* MS39) mushroom was procured from Welkin Overseas Pvt. Ltd. located in Haridwar, Uttarakhand, India (29°46′43.7″ N 77°47′26.8″ E). This strain was developed by the Directorate of Mushroom Research (DMR), Solan, Himachal Pradesh, India (30°55′20.0″ N 77°06′08.9″ E). DMR is a government organization dedicated to the development of mushroom research technologies in India. The healthy spawn of *A. bisporus* grown on sterile wheat grains was stored at 4 °C until its application. The mushroom substrate was prepared using wheat straw (WS) (chopped into 3–5 cm pieces) following a short-term method of composting [39], as recommended by DMR [40,41]. Composting was performed using a traditional pile system facilitated with aeration through percolated plastic pipes. A nitrogen, phosphorus, and potassium (N:P:K) fertilizer treatment of 33:10:25 was used to enrich the substrate with essential nutrients after moistening in tap water. The compost was pasteurized under controlled environmental conditions. For the casing material, the soil was collected from the departmental garden of Gurukula Kangri (Deemed to be University), Haridwar, India (29°55′10.3″ N 78°07′08.3″ E). Sewage sludge was collected in sterile polyethylene bags (5 kg capacity) from Sewage Treatment Plant (27 MLD capacity STP) located at Jagjeetpur, Haridwar, India (29°54′01.2″ N 78°08′14.9″ E).

### 2.2. Experimental Design

**Figure 1:**
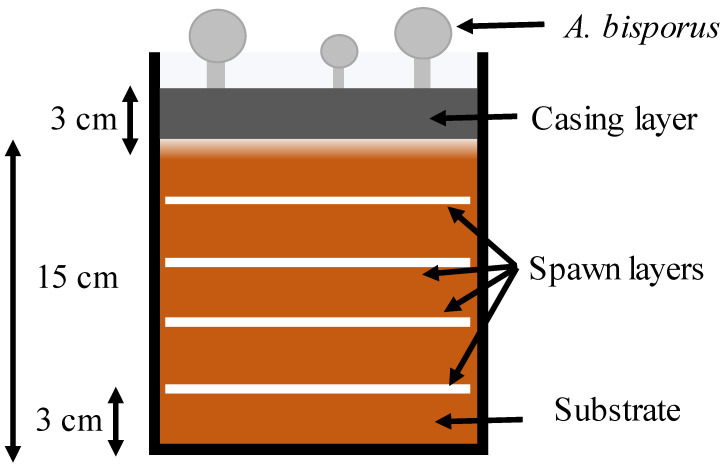
Illustration of each experimental unit for *A. bisporus* cultivation (the bag was 20 cm in diameter).

For the cultivation of *A. bisporus*, a total of 5 kg compost was filled in a sterile polyethylene bag (20 cm diameter). Thirty grams of healthy spawn was divided between four layers as the bag was being filled with compost to a depth of 15 cm (Figure 1). Afterward, the bags were placed in an environmentally controlled cultivation room maintained at 25 °C, relative humidity of 80%, a light intensity of 650 lux, and a CO_2_ concentration of 10,000 ppm that lasted for 20 days. Sewage sludge was sieved through a mesh size of 1300 µm (No. 16; Elysian IN) and sterilized in an autoclave at 121 °C for 20 min (KI- 174, Khera Instrument, IN). There were five different treatment mixtures of loam soil (LS) and SS, i.e., 0 mg/kg (control as absolute LS), 50 g/kg (950 g LS + 50 g SS), 100 g/kg (900 g LS + 100 g SS), 150 g/kg (850 g LS + 150 g SS), and 200 g/kg (800 g LS + 200 g SS). The formulated casing material was treated using a 10% formalin solution followed by air drying for 3 days. The casing material was applied 3 cm thick to the top of the compost in each bag and watered accordingly to maintain 60% moisture content. The pinhead formation was initiated by adjusting the air temperature (12 °C), humidity (75%), light intensity (650 lux), and CO_2_ (900 ppm). Finally, the fruiting bodies of *A. bisporus* were harvested in three subsequent flushes and expressed as total yield. Fruiting bodies were carefully twisted and harvested (after 5–7 days) once they reached a maximum diameter of 3–4 cm.

### 2.3. Analytical Methods

The mushroom substrate and formulated casing materials were analyzed for various physicochemical and metal element parameters following standard methods [8,42]. The substrate samples were analyzed for the selected physiochemical and metal element parameters immediately before the spawn inoculation, while the casing material samples were analyzed before applying it on the substrate layer after achieving a complete spawn run (20th day). Whole fruiting bodies of mushrooms were obtained from each respective treatment and air-dried for further elemental analysis. A 1:10 suspension of dried mushroom substrate or casing material in distilled H_2_O was prepared to analyze pH and electrical conductivity using a calibrated ESICO 1615 m (ESICO, IN). The organic carbon was estimated by following Walkley and Black method [43]. Total nitrogen was estimated by following the digestion and distillation method using Agilent Cary 60 UV-visible spectroscopy. The contents of metal elements viz., Cu, Cr, Cd, Fe, Mn, and Zn were estimated using an Inductively coupled plasma atomic emission spectroscopy (ICP-OES: 7300 DV, Perkin Elmer, Massachusetts, USA) instrument. For the estimation of metal elements, the samples were digested using a di-acid mixture (1:6) of nitric–perchloric acids and then analyzed using an ICP-OES instrument using standardized metal solutions (0, 2.5, 5, 10, 25, 50, and 100 mg/L). Similarly, the harvested mushroom bodies were oven-dried at 105 °C and then converted into fine powder. Mushroom biomass was also analyzed for selected metal elements following the same method of the substrate and expressed as mg/kg dry weight basis (dwt.).

### 2.4. Bioaccumulation Factor and Prediction Modeling of Metal Elements Uptake

The uptake and localization of metal elements by living organisms can be enumerated by analyzing their concentration in the organism’s tissues and substrate/media in which they grow. The bioaccumulation factor (BAF) is a widely used tool that determines the uptake efficiency of organisms. In this study, the BAF of *A. bisporus* for metal elements uptake from its SS amended substrate was calculated using the following formula (Equation (1)):

BAF = C_M_/C_S_ (1)

where C_M_ and C_S_ are the concentrations of the metal element in mushroom fruiting bodies and substrate (wheat straw + SS), respectively.

Multiple linear regression (MLR) modeling is one of the most implemented methods of determining the uptake and accumulation of these elements in different parts of crops. The generalized linear regression method was used to assess the impact of selected physicochemical characteristics of mushroom casing material/substrate and their potential uptake by the fruiting bodies of *A. bisporus*. For this, the following MLR model was constructed to predict the uptake of Cd, Cu, Cr, Fe, Mn, and Zn elements by *A. bisporus* (Equation (2)):

y = β + (β × pH_C_) + (β × OC_C_) + (β × ME_S+C_) (2)

where y indicates the concentration (mg/kg dwt.) of net metal element absorbed by *A. bisporus* during its vegetative growth, β indicates the regression coefficients, pH_C_ and OC_C_ are the pH and organic carbon (mg/kg) of the casing material, while ME_S+C_ indicates the sum of metal elements present in the mushroom substrate and casing material, respectively. Besides this, the constructed models were validated using two different validation tools, viz., model efficiency (ME) and root mean square error (RMSE), as suggested by Eid et al. [44].

### 2.5. Software and Statistics

All experiments were performed in triplicate (*n* = 3 replicates × 5 treatment groups = 15 experimental units) under controlled environmental conditions. The cultivation bags were placed on a vertical plastic rack in a completely randomized design. One sample from each bag was collected, i.e., three for each treatment, and the mean parameter value was used. For the statistical and modeling analyses, Microsoft Excel (2019, Microsoft Corp., Redmond, WA, USA) and OriginPro (2020b; OriginLab Corp., Northampton, MA, USA) software packages were used. The level of statistical significance for analysis of variance (ANOVA) and correlation studies was Prob. (*p*) < F values of 0.05.

## 3. Results and Discussion

### 3.1. Characteristics of the Mushroom Substrate and Casing Materials

The results showed that the WS-based substrate comprised various nutrient parameters, including OC, N, and metal elements. Moreover, the two different casing materials, i.e., loam soil and SS, showed significant variation (*p* < 0.05) in terms of physicochemical and metal element parameters such as pH, EC, OC, TKN, C/N ratio, Cu, Cr, Cd, Fe, Mn, and Zn (Table 1). Moreover, the metal elements and other major nutrient parameters (OC and TKN) were significantly (*p* < 0.05) increased in the casing material after SS mixing when compared to the control treatment (Table 2). The casing material had Fe as the most dominating metal element, followed by Mn, Zn, Cu, Cr, and Cd, respectively. The coefficient of variance (CV) values showed lesser uncertainty in parameter measurement (<2%). Thus, the mixing rate of SS greatly contributed to increasing the levels of various nutrients and metal elements in the casing material. Previously, Eid and coworkers [44] determined the physiochemical and metal element properties of SS and reported that SS had significant levels of various nutrients, which were further useful for increasing the soil fertility under *Abelmoschus esculentus* crop cultivation.

### 3.2. Effects of Sewage Sludge as Casing Material on Yield and Productivity of A. bisporus

The amendment of SS showed increased production of *A. bisporus* as compared to control treatment with no SS application (Table 3). In a total of three harvested flushes, the minimum yield (165.53 g) of *A. bisporus* was observed using control treatment, whereas the maximum yield (195.07 g) was achieved using a 200 g/kg SS amendment rate. As the SS amendment rate increased, the yield also increased significantly (*p* < 0.05). Biological efficiency corresponds to net mushroom yield from the unit dry weight of the substrate; it was the highest (65.02%) for the 200 g/kg SS rate. Mushroom yield and productivity are largely impacted by the type of substrate used for their cultivation along with the suitable environmental and irrigation conditions. Certain additives such as organic manure, chemical fertilizers, nano-fertilizers, and biofertilizers are widely used to increase production [45]. OC and TKN are main nutrient parameters that should be at optimum levels. The enzyme system of fungi helps in the breakdown of carbon-based compounds and utilizes them as their primary energy source. The polysaccharides and the polymer lignin compounds from dead organic wastes breakdowns into simpler carbon molecules, which are further taken by mycelia and help in mushroom growth. Thus, a significant amount of OC is utilized by mushrooms during their vegetative growth phases. Similarly, the optimum level of N is essential to be supplied to obtain an efficient yield and productivity of mushrooms [8]. Apart from the nutrient availability in the substrate, the growth of mushrooms also depends on the casing material on which they form a primordium and start forming fruiting bodies. Therefore, certain physiochemical and nutrient interactions take place between the mycelial network and the casing material [46,47]. The SS composition provided a better structure to the thicker mycelia necessary for the production of *A. bisporus* and did not inhibit its production.

Sewage sludge is a well-established soil amendment for various crops (*Abelmoschus esculentus*, *Brassica oleracea*, etc.) [8,48,49]. However, to date, no study is available on the use of SS as a casing material for mushroom cultivation [50]. Different types of casing materials (clay, sand, loam, Fargo silty, chalk, forest soils, vermiculite Rockwood, peat wool, sawdust, coir pith, wood charcoal, paper sludge waste) have shown promising impacts on mushroom growth and productivity [51]. Khakimov et al. [52] assessed the impact of sierozem, bio-humus, decomposed manure, sawdust, the different proportion of chalk on *A. bisporus* production and found that a combination of sierozem + decomposed manure + bio-humus + chalk gave the highest mushroom yield due to the presence of optimum nutrient levels.

### 3.3. Effects of Sewage Sludge as Casing Material on Metal Elements Accumulation by A. bisporus

**Figure 2:**
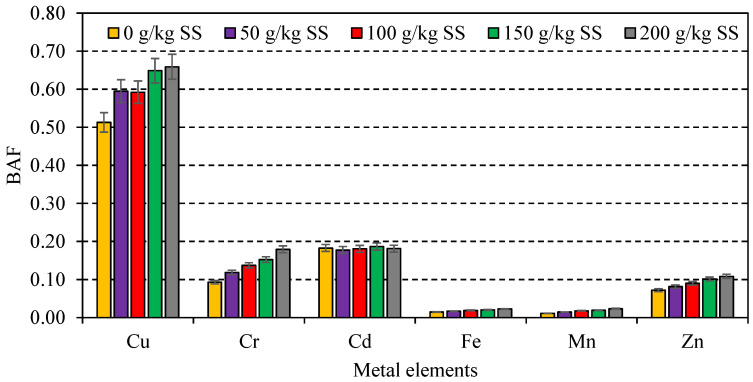
Bioaccumulation factor of metal elements uptake by *A. bisporus* cultivated on sewage sludge-based casing material.

Sewage sludge as an additive of casing material increased the bioaccumulation of various metal elements into the fruiting bodies of *A. bisporus*. In a total of three harvests, the accumulation of selected metal elements (Cu, Cr, Cd, Fe, Mn, and Zn) was significantly (*p* < 0.05) higher in all SS treatments as compared to control treatment (Table 4). However, the first mushroom flush showed slightly higher metal accumulation as compared to the second and third flush, which might be due to the high accumulation efficiency of mushroom mycelia at the early development stage. Moreover, the BAF of selected metal elements was less than 1, which indicated that their levels in the edible fruiting bodies might not have exceeded the toxic limits (Figure 2). The decreasing order of metal elements accumulated by *A. bisporus* was ranged as Fe > Zn > Cu > Mn > Cr > Cd. However, Cd is primarily considered a hazardous metal that can cause severe health hazards in mushroom consumers. As it is a Zn substitute, Cd might become accumulated in mushroom bodies as a competing element if it is available in substrates/casing materials. However, the levels of Cd did not reach high levels as compared with control samples. Other metal elements (Cu, Cr, Fe, Mn, Zn) are essentially taken by fungal hyphae, which play an important role in certain physiological, biochemical, and virulence mechanisms in mushrooms [8].

Despite the nutritional benefits, SS is also known to bear many potential pathogens. Therefore, its proper sterilization should never be omitted. Even if no living organisms are present, the SS may contain mobile genetic elements, such as plasmids and viral particles that strongly bind with charged particles and resist degradation for prolonged periods. Those genetic elements often carry genes for antibiotic resistance and can be passed on to microorganisms present in the soil that was used as a casing material or as a substrate mixture, thus increasing antibiotic resistance in microorganisms that can be regarded as a hazard for human health. Additionally, some microorganisms sporulate and can survive poorly executed sterilization. Therefore, it is highly recommended that the sewage sludge should be fully inactivated before using it as a casing material and monitor the occurrence of certain toxic metals (Cd, As, Pb).

Previous studies have shown that increased levels of different metal elements, including Fe in the casing soil, might be beneficial for higher mushroom production [53]. Similarly, Bhupathi et al. [54] emphasized the role of mineral properties of casing soil on the yield and productivity of milky mushrooms (*Calocyble indica*). They reported that the near-neutral (7.6) pH was more suitable for the rapid fruiting body formation and nutrient accumulation. Recently, the authors of [8] studied the impact of wastewater loading on the growth and productivity of *A. bisporus* mushroom cultivation on WH and sugar cane bagasse substrate and found that the metal elements in mushroom bodies increased as the wastewater concentration increased.

### 3.4. Prediction Models for Evaluating Metal Elements Accumulation by A. bisporus

**Figure 3:**
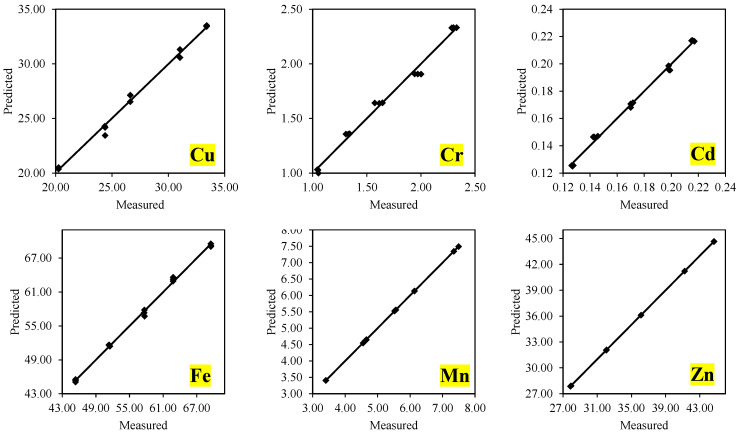
Measured vs. predicted metal elements uptake by *A. bisporus* cultivated on sewage sludge-based casing material.

Prediction modeling is an efficient method of determining the metal element enrichment by various organisms, including crop plants and fungi [44,48]. Multiple linear regression is one of the most efficient among various methods. The present investigation used a three-factor approach for determining the effective metal element uptake by *A. bisporus* grown on WH-substrate and SS amended casing material. The results revealed that the selected independent variables (initial pH, OC, and metal elements) were highly correlated with metal element uptake by *A. bisporus*. However, their affinity toward metal elements varied widely based on their uptake amount, as shown by the estimated regression coefficients (Table 5). More specifically, the pH of casing material was positively correlated for Cu, Fe, and Zn uptake while negatively for Cr, Cd, and Mn elements. On the other hand, the metal elements uptake was positively influenced by the OC of casing material for Cu, Cd, Fe, and Zn while negatively for Cr and Mn uptake, respectively. Similarly, initial metal element availability in both substrate and casing material also affected their uptake positively except for Cu, which showed a negative association. The metal uptake by mushroom mycelia depends on the adsorption and absorption of their specific ions, which are greatly impacted by both pH and OC, as discussed in previous studies [55,56]. Overall, the model equations given in Table 5 can be used for the precise prediction of metal element uptake by *A. bisporus* grown on SS amended casing materials. The models were characterized by a good coefficient of determination (*R*^2^ > 0.9900) high model efficiency (ME > 0.98) and low root means square error (RMSE < 0.410), respectively. Therefore, the models showed acceptable prediction performance when compared to the actual observations as specified in Figure 3.

There are not many studies conducted on the implementation of regression models for metal elements uptake by cultivated *A. bisporus* mushroom. Out of certain recent studies, Kumar et al. [8] constructed the regression and artificial neural network models for metal elements uptake by *A. bisporus* grown on the wheat straw substrate and reported that both methods were efficient in uptake prediction and health risk assessment. Similarly, Indolean et al. [57] also performed laboratory experiments for Cu uptake by *Lactarius piperatus* mushroom and evaluated the uptake process using artificial neural network models. They also reported that the absorption capacity of models was significantly correlated with the initial Cu dose, the weight of substrate used for *L. piperatus* cultivation.

## 4. Conclusions

This study concluded that the SS could be successfully used as a casing amendment for *A. bisporus* cultivation. The bioaccumulation of different metal elements (Cu, Cr, Cd, Fe, Mn, and Zn) increased with an increase in the SS rate from 0 to 200 g/kg. The ICP-OES analysis revealed that the highest bioaccumulation (BAF ≤ 1)) of metal elements was observed using 200 g/kg SS application as casing material. The higher BAF may be alarming due to the high toxicity of some metal elements such as Cd. Thus, it needs to be properly monitored and regulated while using SS as a casing additive. Moreover, the multiple linear regression models of high efficiency were useful to monitor the metal element uptake by *A. bisporus*. This study demonstrated the sustainable use of SS for the formation of mushroom casing material, which might contribute to the emergence of a synergistic agro-economy creation and biosolid management practice. Further studies on large-scale production and biomonitoring of other metal elements uptake by *A. bisporus* are highly recommended.

## Figures and Tables

**Table 1:** Characteristics of the wheat straw-based mushroom substrate, loam soil, and sewage sludge used for *A. bisporus* cultivation.

Parameter	WS-Substrate		Loam Soil (LS)		Sewage Sludge (SS)	
	Value	CV (%)	Value	CV (%)	Value	CV (%)
pH	7.06 ± 0.03	0.37	7.31 ± 0.01	0.16	6.10 ± 0.01	0.16
Electrical conductivity (dS/m)	6.13 ± 0.04	0.60	5.31 ± 0.04	0.75	6.92 ± 0.02	0.30
Organic carbon (g/kg)	494.86 ± 2.86	0.57	10.10 ± 0.61	0.53	122.62 ± 0.42	1.00
TKN (g/kg)	17.26 ± 0.03	0.15	0.56 ± 0.04	7.14	6.44 ± 0.10	1.55
C/N ratio	28.67	-	18.03	-	18.63	-
Cu (mg/kg)	34.43 ± 0.57	1.67	5.07 ± 0.03	0.68	56.03 ± 0.07	0.12
Cr (mg/kg)	8.20 ± 0.12	1.54	2.75 ± 0.01	0.15	10.51 ± 0.03	0.26
Cd (mg/kg)	0.54 ± 0.01	0.56	0.15 ± 0.01	1.31	2.49 ± 0.01	0.24
Fe (mg/kg)	3091.92 ± 0.38	0.01	16.68 ± 0.09	0.52	51.87 ± 0.17	0.32
Mn (mg/kg)	310.73 ± 0.89	0.28	5.80 ± 0.01	0.14	12.06 ± 0.05	0.44
Zn (mg/kg)	385.89 ± 0.80	0.20	3.05 ± 0.01	0.34	117.98 ± 0.45	0.38

Values are mean ± SD of three analyses; CV: coefficient of variance (%); All parameters of LS and SS were significantly different from WS-substrate at *p* < 0.05.

**Table 2:** Physicochemical and metal element characteristics of casing material prepared using loam soil and sewage sludge for *A. bisporus* cultivation.

Characteristics	Sewage Sludge Treatment				
	Control (0 g/kg)	50 g/kg	100 g/kg	150 g/kg	200 g/kg
pH	7.31 ± 0.01	7.19 ± 0.01 a	7.05 ± 0.03 a	6.77 ± 0.02 a	6.50 ± 0.02 a
Electrical conductivity (dS/m)	5.31 ± 0.04	5.61 ± 0.01 a	6.03 ± 0.03 a	6.29 ± 0.03 a	6.51 ± 0.03 a
Organic carbon (g/kg)	10.10 ± 0.61	12.41 ± 0.73 a	14.76 ± 0.58 a	20.15 ± 1.03 a	23.44 ± 0.82 a
TKN (g/kg)	0.56 ± 0.04	0.70 ± 0.05 a	0.84 ± 0.09 a	0.94 ± 0.06 a	1.12 ± 0.10 a
Cu (mg/kg)	5.07 ± 0.03	6.53 ± 1.16 b	10.53 ± 0.16 a	13.40 ± 0.15 a	16.24 ± 0.08 a
Cr (mg/kg)	2.75 ± 0.01	3.24 ± 0.03 a	3.75 ± 0.05 a	4.31 ± 0.06 a	4.80 ± 0.04 a
Cd (mg/kg)	0.15 ± 0.01	0.26 ± 0.01 a	0.40 ± 0.01 a	0.52 ± 0.01 a	0.64 ± 0.01 a
Fe (mg/kg)	16.68 ± 0.09	19.25 ± 0.06 a	21.61 ± 0.26 a	24.32 ± 0.18 a	27.16 ± 0.14 a
Mn (mg/kg)	5.80 ± 0.01	6.40 ± 0.01 a	7.05 ± 0.04 a	7.60 ± 0.02 a	8.21 ± 0.01 a
Zn (mg/kg)	3.05 ± 0.01	8.98 ± 0.03 a	14.79 ± 0.14 a	20.88 ± 0.17 a	26.61 ± 0.12 a

Values are mean ± SD of three analyses; a and b: Significantly different and not different from the control group at *p* < 0.05 and *p* > 0.5.

**Table 3:** Mushroom yield and biological efficiency of *A. bisporus* cultivated on sewage sludge amended casing material.

Parameter	Flush	Sewage Sludge Treatment				
		Control (0 g/kg)	50 g/kg	100 g/kg	150 g/kg	200 g/kg
Mushroom yield (g/kg fresh substrate)	1st	64.32 ± 0.26	72.55 ± 0.28 a	75.75 ± 0.55 a	80.79 ± 0.40 a	76.74 ± 2.24 a
	2nd	57.77 ± 0.35	60.95 ± 0.49 a	65.70 ± 0.22 a	64.83 ± 0.65 a	65.95 ± 1.69 a
	3rd	43.45 ± 0.33	45.99 ± 0.39 a	48.24 ± 0.47 a	46.81 ± 1.47 a	52.39 ± 0.70 a
	Average	55.18 ± 10.67	59.83 ± 13.32 b	63.23 ± 13.92 b	64.14 ± 17.00 b	65.02 ± 12.20 b
	Total	165.53	179.49	189.69	192.43	195.07
Biological efficiency (%)	-	55.18	59.83	63.23	64.14	65.02

Values are mean ± SD of three replicates. a and b: Significantly different and not different from control group at *p* < 0.05 and *p* > 0.5.

**Table 4:** Contents of metal elements accumulated by *A. bisporus* fruiting bodies under different sewage sludge treatments.

Metal Element (mg/kg dwt.)	Flush	Sewage Sludge Treatment				
		Control (0 g/kg)	50 g/kg	100 g/kg	150 g/kg	200 g/kg
Cu	1st	21.52 ± 0.02	24.97 ± 0.01	27.06 ± 0.01	31.12 ± 0.01	33.46 ± 0.01
	2nd	20.28 ± 0.01	24.28 ± 0.06	26.58 ± 0.01	31.01 ± 0.01	33.40 ± 0.01
	3rd	18.97 ± 0.01	23.86 ± 0.01	26.23 ± 0.01	30.94 ± 0.01	33.31 ± 0.01
	Average	20.25 ± 1.27	24.37 ± 0.55 a	26.62 ± 0.41 a	31.02 ± 0.09 a	33.39 ± 0.07 a
Cr	1st	1.07 ± 0.05	1.41 ± 0.01	1.69 ± 0.01	1.93 ± 0.01	2.28 ± 0.01
	2nd	1.00 ± 0.01	1.38 ± 0.01	1.63 ± 0.01	1.91 ± 0.01	2.25 ± 0.01
	3rd	0.98 ± 0.01	1.27 ± 0.01	1.59 ± 0.01	1.87 ± 0.01	2.45 ± 0.01
	Average	1.02 ± 0.01	1.35 ± 0.07 a	1.64 ± 0.05 a	1.90 ± 0.03 a	2.33 ± 0.11 a
Cd	1st	0.13 ± 0.01	0.15 ± 0.01	0.17 ± 0.01	0.20 ± 0.01	0.21 ± 0.01
	2nd	0.12 ± 0.01	0.14 ± 0.01	0.16 ± 0.01	0.19 ± 0.01	0.21 ± 0.01
	3rd	0.12 ± 0.01	0.13 ± 0.01	0.16 ± 0.01	0.19 ± 0.01	0.21 ± 0.01
	Average	0.12 ± 0.01	0.14 ± 0.01 a	0.17 ± 0.01 a	0.19 ± 0.01 a	0.21 ± 0.01 a
Fe	1st	48.77 ± 0.01	52.42 ± 0.33	58.13 ± 0.01	63.71 ± 0.01	70.45 ± 0.04
	2nd	44.28 ± 0.01	51.57 ± 0.22	57.57 ± 0.09	62.50 ± 0.01	69.70 ± 0.02
	3rd	43.06 ± 0.01	50.17 ± 0.06	57.23 ± 0.06	62.13 ± 0.01	68.37 ± 0.01
	Average	45.37 ± 3.01	51.39 ± 1.13 a	57.64 ± 0.45 a	62.78 ± 0.82 a	69.51 ± 1.05 a
Mn	1st	3.53 ± 0.01	4.76 ± 0.04	5.58 ± 0.01	6.14 ± 0.01	7.53 ± 0.25
	2nd	3.40 ± 0.01	4.64 ± 0.01	5.59 ± 0.05	6.13 ± 0.01	7.34 ± 0.01
	3rd	3.28 ± 0.01	4.41 ± 0.17	5.46 ± 0.11	6.12 ± 0.01	7.30 ± 0.01
	Average	3.40 ± 0.12	4.60 ± 0.20 a	5.54 ± 0.07 a	6.13 ± 0.01 a	7.39 ± 0.12 a
Zn	1st	30.28 ± 0.08	32.52 ± 0.03	36.39 ± 0.01	41.81 ± 0.01	45.15 ± 0.01
	2nd	27.29 ± 0.05	32.21 ± 0.01	36.11 ± 0.01	41.00 ± 0.01	44.68 ± 0.01
	3rd	26.13 ± 0.01	31.47 ± 0.02	35.86 ± 0.01	40.82 ± 0.01	44.11 ± 0.01
	Average	27.90 ± 2.14	32.07 ± 0.54 a	36.12 ± 0.26 a	41.21 ± 0.52 a	44.65 ± 0.52 a

Values are mean ± SD of three replicates; a: Significantly different from the average value of control group at *p* < 0.05.

**Table 5:** Regression models and their validation results for metal elements uptake by *A. bisporus* cultivated on sewage sludge amended casing material.

Metal Element	Model Equation	*R* ^2^	ME	RMSE	F	*p*
Cu	y = 20.217 + (0.067 × pH_C_) + (1.276 × OC_C_) − (0.360 × ME_S__+C_)	0.9918	0.98	0.375	588.47	<0.001
Cr	y = −2.814 − (0.335 × pH_C_) − (0.017 × OC_C_) + (0.593 × ME_S+C_)	0.9900	0.99	0.044	375.89	<0.001
Cd	y = 0.079 − (0.003 × pH_C_) + (0.006 × OC_C_) + (0.004 × ME_S+C_)	0.9956	0.99	0.004	833.15	<0.001
Fe	y = −6963.721 + (4.645 × pH_C_) + (0.313 × OC_C_) + (2.242 × ME_S+C_)	0.9976	0.98	0.410	1542.49	<0.001
Mn	y = −906.571 − (0.096 × pH_C_) − (0.237 × OC_C_) + (2.885 × ME_S+C_)	0.9924	0.98	0.003	479.34	<0.001
Zn	y = −63.503 + (0.058 × pH_C_) + (0.891 × OC_C_) + (0.208 × ME_S+C_)	0.9988	0.99	0.002	323.58	<0.001

pH_C_: pH of casing material; OC_C_: organic carbon of casing material; ME_S+C_: metal element concentration in substrate + casing material; ME: model efficiency; RMSE: root mean square error.

## Data Availability

Not applicable.
